# High Readmission Rate in a Patient With Chronic Heart Failure, End-Stage Renal Disease, and Chronic Pulmonary Obstructive Disease

**DOI:** 10.7759/cureus.97263

**Published:** 2025-11-19

**Authors:** Akila B Jayanthan, Peter DesRochers, Sabrina Shafi, Roxana Lazarescu

**Affiliations:** 1 Internal Medicine, Saba University School of Medicine, The Bottom, BES; 2 Internal Medicine, Medical University of the Americas, Potworks, KNA; 3 Internal Medicine, New York Institute of Technology College of Osteopathic Medicine, Old Westbury, USA; 4 Internal Medicine, Wyckoff Heights Medical Center, New York, USA

**Keywords:** chf exacerbation, chronic heart failure (chf), congestive heart failure (chf), copd: chronic obstructive pulmonary disease, end-stage renal disease (esrd)

## Abstract

Hospital readmission is common for patients with multiple chronic comorbidities, but it is a burden to both patients and healthcare systems. While this is a multifactorial issue, it is important to identify some of the reasons that contribute to high readmission rates and advocate for concrete interventions that can be implemented. We report the case of a 68-year-old female with congestive heart failure with severely reduced ejection fraction, chronic obstructive pulmonary disease requiring home oxygen, and end-stage renal disease on hemodialysis who was readmitted more than 30 times in the previous year. In the last readmission, the patient was admitted for acute on chronic respiratory failure, which improved with hemodialysis during the hospital stay. Beyond her complex comorbidities, the patient’s recurrent admissions were further complicated by restricted mobility, limited home health aid hours, and treatment noncompliance. Strategies such as consistent professional interpreter use, patient-specific medical education, pharmacy delivery programs, and medically adapted transportation may help reduce hospitalizations. The causes of high readmission rates are multifactorial. This case highlights numerous techniques to increase patient compliance and thereby reduce readmissions. Patient-centered strategies, combined with system-level improvements, are needed to address the complexities faced by patients with multiple comorbidities. Further research is needed to identify additional strategies that reduce readmissions, improve quality of life, and lower morbidity and mortality.

## Introduction

Hospital readmissions for management of chronic illness symptoms and complications pose a significant challenge for both healthcare systems and patients. In the United States, 10% of hospitalizations are readmissions, which contribute to 125,000 preventable deaths and $289 billion of avoidable costs each year [1]. Patients with chronic illnesses are particularly vulnerable, and each readmission increases the risk of nosocomial infections and functional decline that may persist for months after the hospitalization [2].

Despite advances in medical management, congestive heart failure (CHF) is still a leading cause of hospitalization, with 18.2% readmissions within 30 days and 31.2% within 90 days [3]. The burden is even greater when CHF coexists with other chronic illnesses such as chronic obstructive pulmonary disease (COPD) or end-stage renal disease (ESRD). 

Communication barriers and limited adherence to complex medication regimens and demanding dialysis sessions further complicate care and place patients at high risk for readmission. More than 42% of the 60 million Americans who speak more than just English have limited English proficiency (LEP), are less adherent to treatment, and have more complications compared with English-speaking patients [4]. Medication nonadherence is common in chronic illness, with one study estimating that 30%-50% of medications are not taken as prescribed [5].

This report describes a patient with CHF, COPD, and ESRD on hemodialysis who was readmitted to the hospital nearly every other week over the course of a year. Her case highlights how overlapping medical and logistical barriers can drive recurrent hospitalizations and underscores the need for multifaceted, system-level approaches to reduce morbidity, mortality, and healthcare costs in patients with advanced comorbidities.

## Case presentation

In this case, we present a 68-year-old bilingual female with a history of coronary artery disease status post-coronary artery bypass grafting. She has CHF with a left ventricular ejection fraction of 15%-20%. She also has COPD, for which she is on 3 L/min home oxygen, and ESRD requiring hemodialysis three times a week. Furthermore, her history also includes peripheral arterial disease with bilateral stents, abdominal aortic aneurysm status post-graft repair, renal artery stenosis status post-left stent, hypertension, type 2 diabetes mellitus, and hypothyroidism. The patient could communicate in English, but Spanish interpreters were intermittently used during hospitalizations. 

She most recently presented to the emergency department for acute shortness of breath that had worsened over 12 hours. Vitals showed normothermia with a temperature of 97.9°F, hypertension with a blood pressure of 218/136 mmHg, tachycardia with a heart rate of 117 bpm, and tachypnea with a respiratory rate of 29 breaths/min. The patient was initially on continuous positive airway pressure with oxygen saturation of 100%, then transitioned to bi-level intermittent positive pressure (BiPAP). She denied fever, chills, or chest pain. She reported adherence to her medications and dialysis but had missed her dialysis session earlier that day due to respiratory distress. She denied any use of alcohol, tobacco, or illicit drugs. Notably, the patient had been hospitalized one week earlier for similar symptoms with a three-day length of stay, and had over 30 admissions over the last year for similar complaints.

Although clinical suspicion for infection was low, two doses of empiric ceftriaxone were given due to the patient’s tachycardia and tachypnea. A 500-mg dose of IV azithromycin was also administered for atypical organism coverage. Blood pressure improved with BiPAP support and IV magnesium sulfate.

Physical examination revealed diffuse wheezing and basilar crackles, but no peripheral edema or calf tenderness. Laboratory evaluation demonstrated elevated lactate of 3.9 mmol/L, procalcitonin of 1.8 ng/mL (0.05-0.09 ng/mL), but no leukocytosis or acidosis. B-type natriuretic peptide was >5000 pg/mL, consistent with the patient’s baseline. Troponin was negative on arrival and peaked at 68.5 ng/L four hours later, without clinical concern for acute coronary syndrome. Tests for COVID-19, influenza A, influenza B, and respiratory syncytial virus were negative. Chest X-ray was consistent with previous imaging, showing diffuse interstitial septal thickening consistent with pulmonary edema, mild patchy peribronchial cuffing, a moderate left-sided pleural effusion with adjacent airspace disease, and no cardiomegaly. A repeat echocardiogram showed no change from two months before, with a left ventricular ejection fraction of 15%-20%.

The patient was admitted for acute on chronic respiratory failure likely due to COPD exacerbation or volume overload in the setting of CHF. Treatment included IV diuretics, corticosteroids, bronchodilators, and continuation of home medications, including sacubitril/valsartan, carvedilol, and hydralazine. Intake and output, blood pressure, and glucose levels were closely monitored throughout the admission. The patient received hemodialysis on hospital days 1 and 3, which resulted in significant improvement in her respiratory symptoms. Interventional radiology was consulted for thoracentesis for the pleural effusion, but the patient declined.

By hospital day 2, the patient was in no acute distress and comfortable on a nasal cannula with 3 L oxygen. She was discharged home on hospital day 3. The patient was encouraged to follow up with her primary care provider within one week, and she was given a referral to home pulmonary rehabilitation to support long-term respiratory function and reduce the risk of future hospital readmissions. 

## Discussion

In this case, we described a patient with multiple comorbidities, including CHF with severely reduced ejection fraction, COPD with home oxygen dependence, and renal failure requiring hemodialysis, who has been readmitted more than every other week on average over the past year. This high readmission rate illustrates the difficulty of managing complex chronic illnesses. For patients, recurrent hospitalizations increase the risk for nosocomial infections and contribute to psychological distress. Furthermore, patients become deconditioned during hospitalizations due to limited mobility, sleep deficit, and malnutrition, which can lead to a 2%-5% loss of muscle mass each day a patient is bedbound, and a 15.8% risk of readmission [2]. For a 150 lb patient with muscle mass accounting for 30% of body weight, a 2% loss of muscle mass is equivalent to 1 lb a day. For the healthcare system, frequent readmissions put a strain on already-limited resources and can contribute to provider frustration. There is also a risk for cognitive biases as providers anchor onto the diagnoses that a patient is typically readmitted for. Thus, reducing readmissions is critical for both patients and providers. Although no single solution is likely to resolve these challenges, examining what has already been done for this patient and what additional strategies could be implemented helps identify both patient-specific opportunities and broader systemic gaps.

Language barrier

One factor complicating this patient’s care was communication. Although this bilingual patient was comfortable having conversations with the medical team in English, the team often explained her care in both English and Spanish. Language proficiency is a spectrum, so being comfortable with conversational English does not mean comfort with complex medical discussions, and both patients and providers overestimate language proficiency [6]. Therefore, offering interpretation into the patient’s preferred language should be standard for all clinical conversations. One caveat is that patients may not request clarification or interpretation out of politeness, to avoid delaying care, or because of embarrassment about their level of English proficiency [7]. This makes it especially important to continuously check for patient understanding, for example, by having the patient repeat key points from what was discussed.

Despite the need, interpreters are often underutilized due to inconvenience. First, the availability of in-person interpreters is limited, and connecting with telephonic or video interpreters is a lengthy process. Second, there are technical difficulties such as poor internet or cellular connection and background noise from hospital machines, staff, and other patients. Third, an ill patient may speak softly or have hearing difficulties, making it difficult for the interpreter to hear the patient and vice versa. These limitations with interpreters lead hospital staff to instead rely on translation apps such as Google Translate (Google Inc., Mountain View, CA, USA) and MediBabble (MediBabble, San Francisco, CA, USA), but these tools are inconsistent and unsafe for use in medical decision-making [8]. Additionally, research shows that patients with LEP are at higher risk for readmission, but that professional interpreter use is associated with reduced readmissions and improved outcomes [4]. To promote good communication, medical teams should be educated on the value of using interpreters consistently, and hospitals should increase access to in-person interpreters. Technology improvements such as noise-reducing microphones, external speakers, and streamlined connection processes would also make telephonic interpreter use more practical. 

In this patient’s case, the medical team used interpreters as needed to emphasize during each admission the risks of inconsistent adherence to medical management, dialysis, and follow-up appointments. 

Medication noncompliance

Another major factor complicating this patient’s care was medication nonadherence. Medications for heart failure, COPD, and ESRD-related complications are intended not only to treat symptoms but also to prevent acute exacerbations, disease progression, and complications. Furthermore, consistent adherence to medical therapy is also a prerequisite for advanced therapies such as transplantation.

Patients may take medications inconsistently or not at all for a variety of reasons, including unfavorable side effects, regimen complexity, cost, difficulty collecting medications, forgetfulness, and lack of perceived need for treatment [5]. Involving family members in medication routines can help provide external accountability, and the patient’s family was at times involved in counseling for this reason. Another approach to increasing medication compliance is patient-specific education that goes beyond general reminders. Studies have shown that tailored education, particularly when paired with counseling and follow-up, improves adherence and reduces adverse outcomes [1]. For example, urging patients to contact their healthcare providers if they experience intolerable side effects or insurance barriers is more effective than broadly reminding patients that medications are important for managing their chronic illnesses.

Difficulty filling prescriptions can also contribute to inconsistent treatment compliance after discharge. For this, there are services such as “meds-to-beds” that deliver medications to the hospital bedside so that patients who are still recovering and unable to visit the pharmacy can leave with their medications in hand. This service has been associated with lower 30-day readmission rates [9]. Similarly, pharmacy home-delivery services have been shown to improve adherence and reduce readmissions in high-risk populations [5]. Interprofessional collaboration adds yet another layer of support as pharmacist-led counseling and reminders are associated with higher adherence in complex patients [10].

Finally, some nonadherence is due simply to forgetfulness. Practical tools can address this problem, including pill bottles that electronically log the last time they were opened and smartphone apps that remind patients to take their medication. Reviewing these strategies with patients who have multiple comorbidities and complex medication regimens can help tailor support to their individual needs. 

Dialysis and follow-up appointments

Regular attendance of dialysis sessions and follow-up appointments is also critical for the management of chronic illnesses. For patients with ESRD, attending hemodialysis regularly is essential. Unfortunately, dialysis takes several hours multiple times a week and often leaves patients severely fatigued, which patients cite as one of the most burdensome aspects of the treatment [11]. This discourages patients from attending dialysis regularly. The patient in this case, for example, told the medical team that she skipped dialysis because the sessions left her feeling weak and made it difficult to take her medications. Missed dialysis sessions are associated with fluid overload and higher rates of hospitalization and mortality [12]. For patients with comorbid CHF, adherence to dialysis also helps prevent volume overload and hospitalizations for decompensated heart failure. Our patient’s severely reduced ejection fraction of 15%-20% was unlikely to be managed by dialysis alone, but it was still an important part of her care.

Timely outpatient follow-up after hospital discharge is also valuable. Studies show that patients with CHF who attend follow-up visits within seven days have significantly lower readmission rates [13]. However, there are obstacles to attending clinic appointments. Transportation barriers are common in patients with advanced comorbidities [14], particularly in urban settings such as Brooklyn, NY. Even with rideshare services, medical appointments are commonly missed, partly because they are insufficient for patients who have mobility limitations requiring assistance [15]. In this case, our patient required assistance to ambulate safely, but she only had a home health aid for a limited time each day. Possible solutions include partnering with community rideshare programs to adapt for medical visits, arranging medical transport services covered by insurance, and using telehealth services for follow-up appointments when clinically appropriate. 

**Figure 1:**
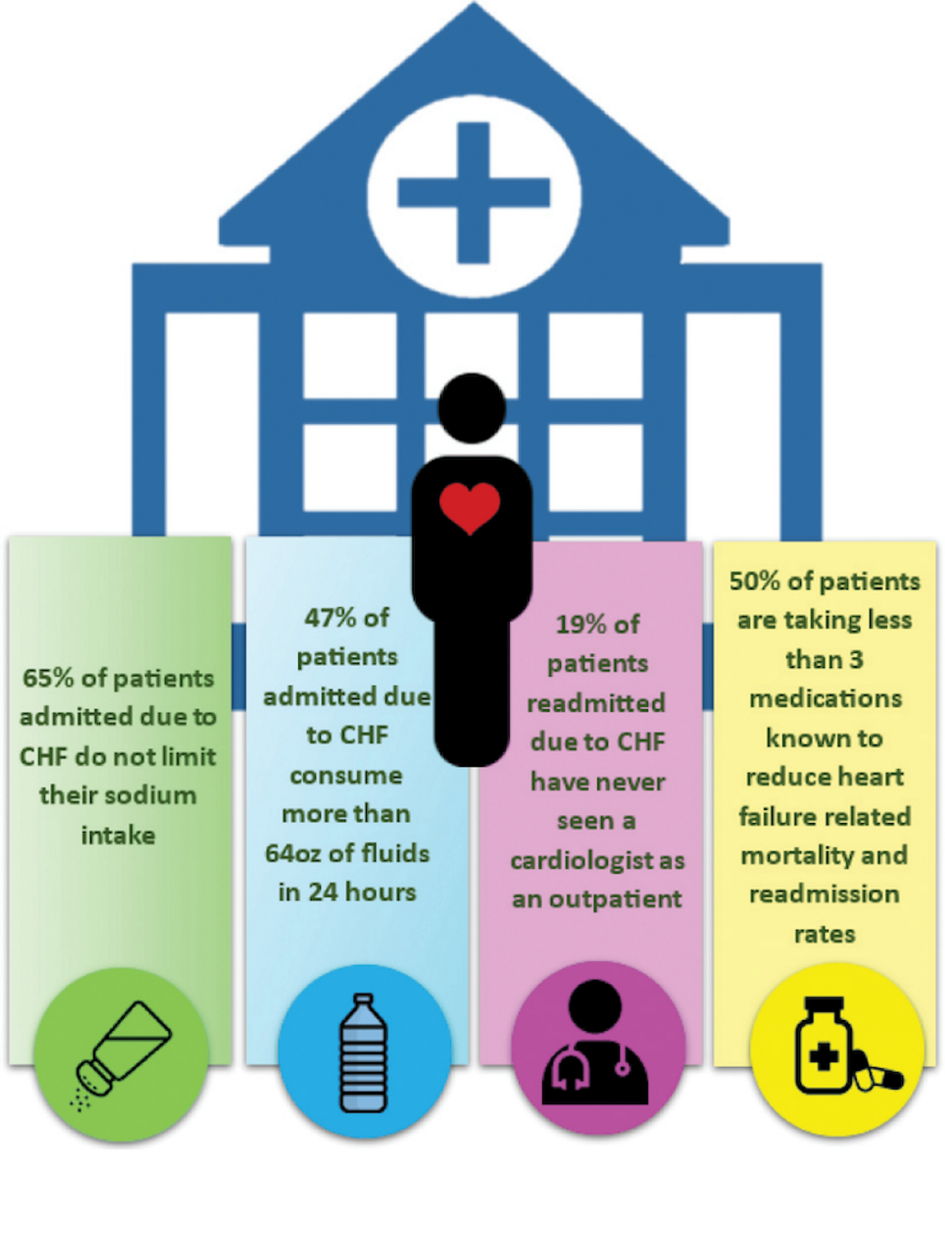
Some causes of CHF readmission in patients CHF, congestive heart failure.

In summary, this case highlights how language barriers and inconsistent treatment compliance contribute to frequent readmissions in medically complex patients. Figure 1 also provides more statistical information on CHF readmission. Addressing these issues requires a multifaceted approach at the level of individual patients and at a system level. While no single strategy is sufficient on its own, integrating these measures can help reduce hospitalizations and improve the quality of life for patients.

## Conclusions

This case illustrates how language barriers, medication nonadherence, and missed dialysis and follow-up appointments contribute to frequent hospital readmissions in patients with complex chronic illnesses. While targeted interventions such as consistent use of professional interpreters, pharmacy collaboration, and medically adapted transportation can address some of these challenges, no single strategy is sufficient on its own. A multifaceted approach that integrates patient-level support with system-level resources is essential to reducing hospitalizations and improving outcomes. Further investigation is needed to identify additional ways the healthcare system can better support high-risk patients in order to decrease morbidity and mortality.
